# The Five Diaphragms in Osteopathic Manipulative Medicine: Myofascial Relationships, Part 1

**DOI:** 10.7759/cureus.7794

**Published:** 2020-04-23

**Authors:** Bruno Bordoni

**Affiliations:** 1 Physical Medicine and Rehabilitation, Foundation Don Carlo Gnocchi, Milan, ITA

**Keywords:** diaphragm, osteopathic, fascia, myofascial, fascintegrity, physiotherapy

## Abstract

Working on the diaphragm muscle and the connected diaphragms is part of the respiratory-circulatory osteopathic model. The breath allows the free movement of body fluids and according to the concept of this model, the patient's health is preserved thanks to the cleaning of the tissues by means of the movement of the fluids (blood, lymph). The respiratory muscle has several systemic connections and multiple functions. The founder of osteopathic medicine emphasized the importance of the thoracic diaphragm and body health. The five diaphragms (tentorium cerebelli, tongue, thoracic outlet, thoracic diaphragm and pelvic floor) represent an important tool for the osteopath to evaluate and find a treatment strategy with the ultimate goal of patient well-being. The two articles highlight the most up-to-date scientific information on the myofascial continuum for the first time. Knowledge of myofascial connections is the basis for understanding the importance of the five diaphragms in osteopathic medicine. In this first part, the article reviews the systemic myofascial posterolateral relationships of the respiratory diaphragm; in the second I will deal with the myofascial anterolateral myofascial connections.

## Introduction and background

Osteopathic manual medicine (OMM) was founded by Dr AT Still in the late nineteenth century in America [1]. OMM provides five models for the clinical approach to the patient, which act as an anatomy physiological framework and, at the same time, can be a starting point for the best healing strategy [1]. The five models are: biomechanical-structural; respiratory-circulatory; neurological; metabolic-nutritional; behavioral-biopsychosocial [1]. The models were developed by the Educational Council on Osteopathic Principles (ECOP) of the American Association of Colleges of Osteopathic Medicine (AACOM) since 1970 and approved by the council of deans in 1987 [1]. In 2012 the five models were also recognized by the osteopathic international alliance (OIA) [1].

In clinical practice there will always be a subjectivization in the choice of the model, with interchangeability of the same models; the models are not limits but rather non-binding reference points [1]. The biomechanical-structural model is a somatic evaluation/approach for body movement and posture; the neurological model evaluates and works on the communication of the various neurological systems. The metabolic-nutritional model highlights how cellular metabolism works, as well as the assimilation of nutrients, the immune and reproductive systems; the behavioural-biopsychosocial model controls and regulates the circadian rhythms of sleep, daily behavior with respect to physical activity, food choice and lifestyle in general [1]. The respiratory-circulatory model, the theme linked to the vision of the five diaphragms, takes into account the homeostasis of the extracellular and intracellular environment with the aim of ensuring that no obstacles prevent either the supply of oxygen and nutrients, or the elimination of cell metabolism waste [1].

The rationale of the respiratory-circulatory model is based on the fact that body fluids must have the ability to circulate freely and the OMM approach will be oriented towards those anatomical structures that can facilitate the objective, including the respiratory diaphragm muscle [1]. The first osteopath who spoke of the treatment of the three diaphragms was Viola Frymann (diaphragm, tentorium cerebelli, and pelvic floor) in 1968 [2]. From the anatomical point of view, a diaphragm is considered as such on the basis of its "horizontal" body position [3]. In reality, in the living, there are no such demarcated and linear structures on one plane, since each macroscopic (and microscopic) structure is three-dimensional, involving infinite axes and planes of movement, with different tissues that influence each other [4-5].

Talking about diaphragm and diaphragms is a convention. In the late seventies, another osteopath made palpatory observations involving four diaphragms. Dr. Gordon Zink evaluated the position of the respiratory diaphragm, the thoracic outlet or high thoracic diaphragm, the pelvic floor or pelvic diaphragm, and the tentorium of the cerebellum or cerebellar diaphragm. Its assessment was based on movements induced by the operator, to look for any restrictions on rotation; while inducing a movement, if the tissue presents an abnormal tension, the slight push of the operator is slowed down by the tissue or anatomical area taken into consideration during the evaluation [1]. By comparing the different assessed diaphragms, the osteopath decides which anatomical structure needs more attention, depending on the greater restriction of palpated movement. In daily clinical practice, a restriction is referred to as "inspiration restriction" or "internal rotation", if one or more palpated diaphragms have a preferentially caudal attitude [1, 6]. On the contrary, a restriction found palpatory in "expiratory restriction" or "external rotation" is called if one or more palpated diaphragms have a preferentially cranial attitude [1, 6]. In 2013, the first scientific article came out which highlighted the relationship of the diaphragm muscle with an additional diaphragm (the fifth), that is, the tongue, through fascial and neurological connections [7]. In 2015 a second article was published on the five diaphragms, with the hypothesis of manual treatment; in 2019 the first clinical article appeared with the five diaphragms and OMM [2, 8]. The article briefly reviews the anatomy of the body diaphragms and the myofascial systemic anterolateral relationships of the same diaphragms.

## Review

Anatomy of the respiratory diaphragm

**Figure 1:**
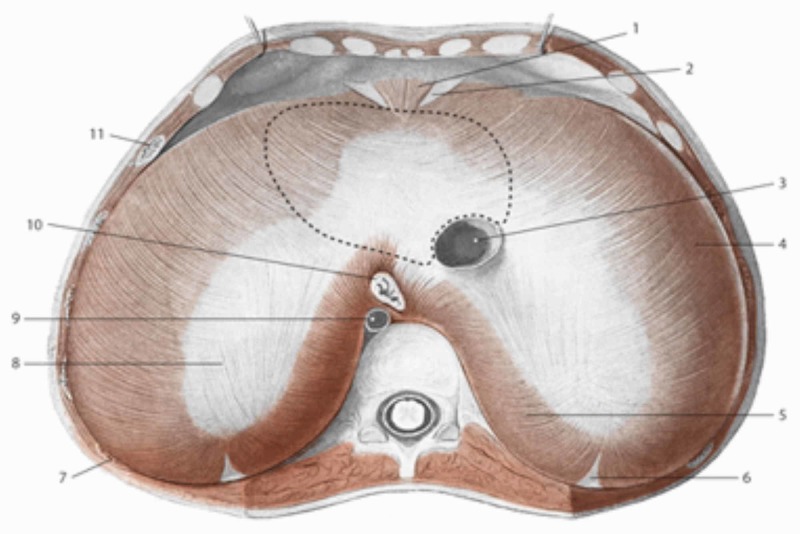
The area above the diaphragm: the dotted line for the support of heart 3: inferior vena cava; 10: esophagus; 9: aorta; 8: tendinous center; 5: lumbar area Reproduced with permission, from Anastasi G, et al., Anatomia dell’uomo, fourth edition [Human Anatomy], 2010, Milan: Edi-Ermes, p 173.

The diaphragm muscle is the main respiratory muscle. The sternal portion of the diaphragm involves the xiphoid process of the sternum from its posterior area with small bundles of fibers [9]. The rib portion arises from the posterior and upper area of the last six ribs, merging with the transverse muscle of the abdomen [9]. The diaphragm involves the dorsal (D11-D12) and lumbar (L1-L4) vertebrae. The diaphragmatic portion of the dorso-lumbar area consists of the right and left medial pillars (MPs), deep and posterior to the sternal and costal (anterolateral) portion; MPs form an "eight" with anterior inclination, for the passage of the abdominal aorta and then of the esophagus [10]. The right medial pillar is longer and wider (D11-L4), while the left medial pillar is shorter and thinner (D11-L2) [9-10]. The vena cava passes through the phrenic center at about D11 [9]. The intermediate pillars with the medial pillars form the medial arcuate ligaments, while the lateral pillars will form the median and lateral arcuate ligaments involving the vertebral body of L1 and L2 and the transverse process, as well as the transverse process with the last rib, respectively [7]. The median arcuate ligament will merge with the large psoas muscle and the lateral arcuate ligament with the quadratus lumborum muscle [7]. The azygos root and sympathetic nerves pass between the medial and intermediate right pillars, while the hemizygos vein and left sympathetic nerves pass between the medial and intermediate left pillars; at the level aortic orifice is located inferiorly of the cistern Quilo (Figure 1) [7].

**Figure 2:**
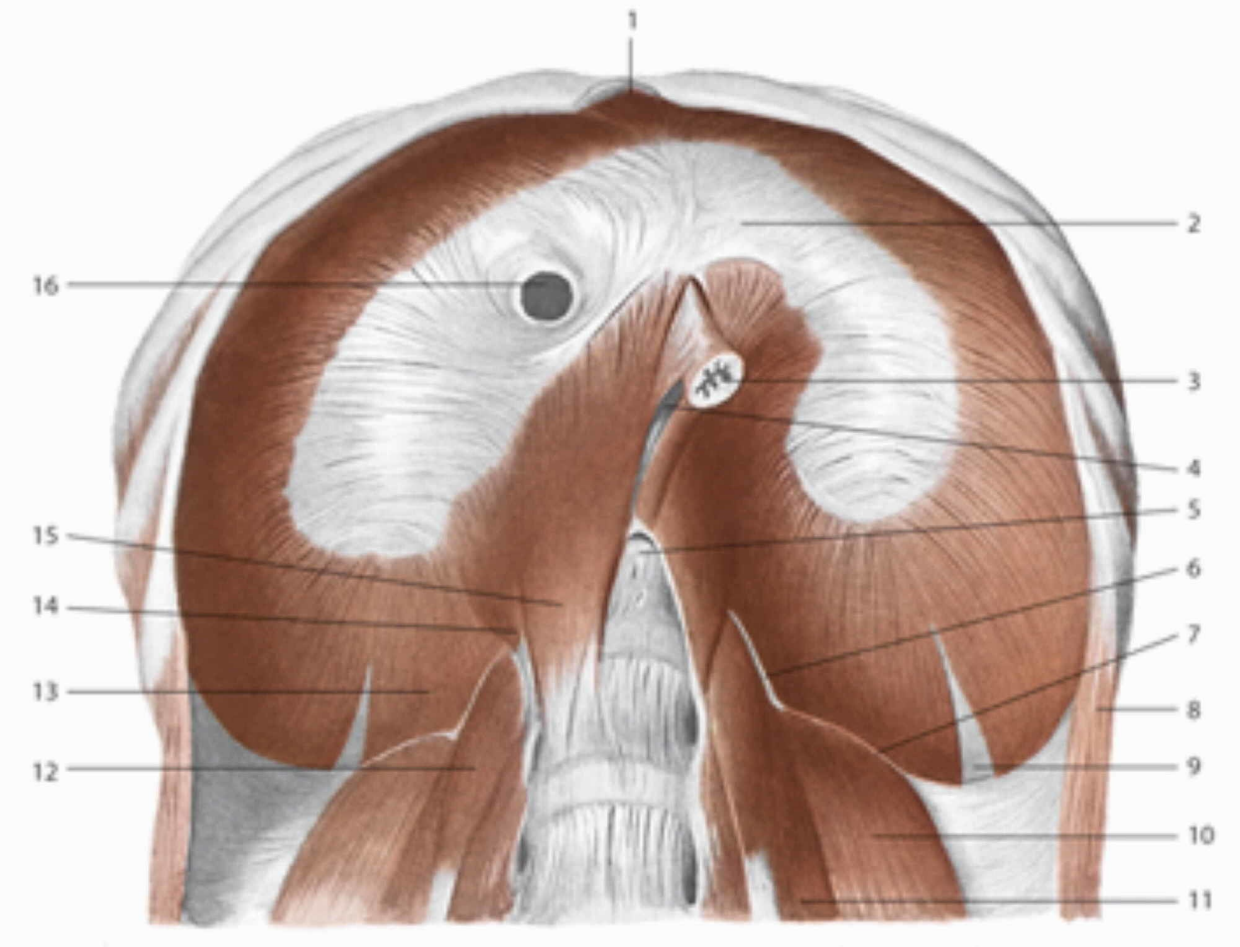
The sub-diaphragmatic area 2: tendinous center or phrenic; 16: inferior vena cava; 3: esophagus; 5: aortic orifice; 15: medial pillar; 14: intermediate pillar; 13: pillar lateral; 6: pillar arcuate medial; 7: lateral arcuate ligament; 10: quadratus lumborum muscle; 11: psoas major muscle. Reproduced with permission, from Anastasi G, et al., Anatomia dell’uomo, fourth edition [Human Anatomy], 2010, Milan: Edi-Ermes, p. 172.

The innervation of the diaphragm muscle comes from the left and right phrenic nerves as well as the vagus nerves; the latter involves the portion of the esophagal hiatus (Figure 2) [7].

Anatomy of the pelvic floor

The pelvic diaphragm, first named in 1861, is made up of the levator ani muscle (made up of the iliococcygeus, pubococcygeus and puborectal muscles), and a second muscle referred to as the coccygeal or ischiococcygeal [11]. The triangular ligament (or urogenital diaphragm or Carcassonne fascia or middle perineal aponeurosis) is the most caudal portion of the pelvic floor, placed externally and horizontally [11-12]. The triangular ligament includes the deep transverse muscle of the perineum; the anterior portion of the ligament is crossed by the urinary and genital ducts, stretched between the two ischio-pubic branches and turned with the apex towards the pubic symphysis [11-12]. The levator ani extends from the internal surface of the pubis to the side of the symphysis, up to the ischial spine; in its path, it involves the internal obturator muscle with a myofascial connection (tendon arch of the levator ani). The pubococcygeus and puborectal muscle form the anococcygeal ligament posteriorly; the iliococcygeal portion originates from the tendon arch of the levator ani, moving medially up to the coccyx (forming a connective raphe or anococcygeal ligament) [11-12]. The ischiococcygeal muscle constitutes the posterosuperior portion of the pelvic diaphragm and originates from the lateral margin of the last sacral segments and coccyx, ending with a thinned portion on the ischial spine and on the neighbouring portion of the sacrospinous ligament [11-12]. The upper portion of the pelvic floor is covered with the endopelvic fascia. Another muscle is an integral part of the pelvic muscle complex, that is, the gluteus maximus. The gluteus maximus muscle is connected via a connective tissue strip at the level of the ischioanalis fossa to the muscle complex of the levator ani; a contraction of the latter is equivalent to a contraction of the gluteus maximus, as demonstrated by electromyographic and magnetic resonance assessments [13]. The innervation of the pelvic musculature is complex, as smooth muscle fibers (not just skeletal muscle fibers) can be found in the levator ani muscle group; these smooth fibers are found mainly in the central and medial area [14]. This area rich in smooth muscle fibers is innervated by the sympathetic nerves of the inferior hypogastric plexus; the remaining muscular area of the pelvic floor is innervated by the levator ani nerve and the pudendal nerve [15].

Anatomy of the thoracic outlet

Anatomists define the thoracic opening or upper thoracic diaphragm as a thoracic inlet, giving the motivation that it is an orifice where air and food passes; for clinicians, it is defined as thoracic outlet, as the passage of blood vessels is emphasized [16]. The upper thoracic diaphragm consists of the sternal bone and the joints between the first two ribs and the clavicle; the clavicle and the first two ribs will involve the scapula and the first two thoracic vertebrae, respectively [16]. The muscle components are the trapezius muscle, the three scalene muscles, the subclavian muscle and the pectoralis minor muscle; to remember, the presence of the scalene minimus muscle, which may contain a muscular or purely connective structure [17]. Different vascular-nervous and visceral structures cross the area. The lower roots and trunks of the brachial plexus with the artery and subclavian vein cross the muscular space, between the anterior and middle scalene, with the base consisting of the first rib; this passage is defined as an interscalene triangle [16]. The nervous and vascular pathways continue, to cross a space between the clavicle and the first rib (costoclavicular space) [16]. The sub-coracoid tunnel, below the tendon of the pectoralis minor muscle, is the third passage portion of the vascular-nervous package [16]. Inside the upper thoracic diaphragm, we find the pleural dome and the Sibson fascia [18].

Anatomy of the tongue

The lingual complex is made up of intrinsic and extrinsic muscles. The intrinsic muscles are: transversalis, verticalis, inferior longitudinalis and superior longitudinalis [19]. The extrinsic muscles, unlike the previous ones, involve the mandibular bone (in particular the genioglossus muscle) and the hyoid bone: genioglossus, styloglossus, hyoglossus and palatoglossus. The intrinsic and extrinsic muscles are in pairs (right and left), for a total of 16 muscles [19]. Some authors consider two other muscles forming the lingual complex, which are part of the extrinsic musculature: glossopharyngeus and chondroglossus. The first is a muscle strip from the superior pharyngeal constrictor muscle, while the second is a small contractile district, which derives from the hyoglossus muscle [19]. The innervation of the tongue comes from the lingual nerve and the hypoglossal nerve; the two nerves communicate before reaching the lingual complex [20]. The hypoglossal nerve with the lateral and medial branches enters the ventrolateral area of the tongue and innervates the genioglossus muscle of the right and left (in its posterior portion), building a cross innervation [21].

Anatomy of tentorium cerebelli

**Figure 3:**
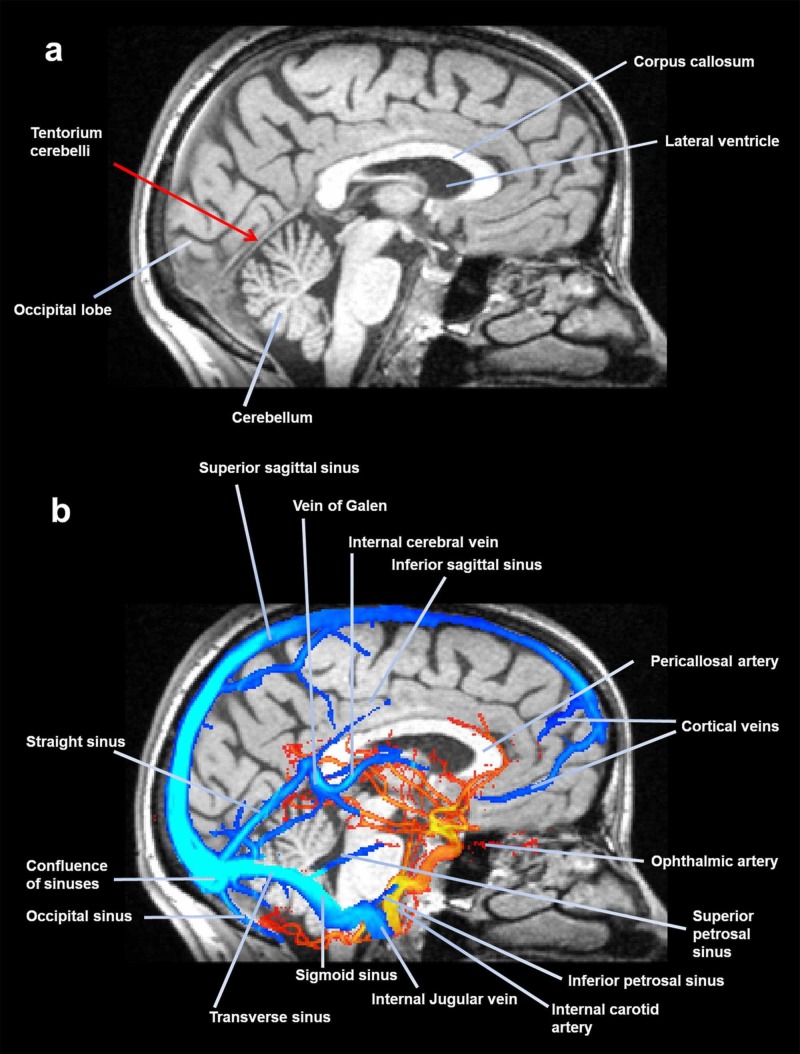
A midsagittal slice of magnetic resonance, where the tentorium is visible. The image shows (above) some portions of the meninges, while the following image (below) shows the venous sinuses and some arteries. Figure A shows the tentorium cerebelli, occipital bone, corpus callosum, lateral ventricle and cerebellum. Figure B shows the superior sagital sinus, vein of Galen, internal cerebral vein, inferior sagital sinus, pericallosal artery, cortical veins, straight sinus, ophthalmic artery, confluence of sinuses, superior petrosal sinus, occipital sinus, inferior petrosal sinus, internal carotid artery, internal jugular vein, sigmoid sinus and transverse sinus. Image reproduced with the permission of Marrcella Maria Laganà PhD, Radiology, IRCCS Fondazione Don Carlo Gnocchi Onlus, Milan, Italy.

The tentorium cerebelli is the posterior portion of the complex of reciprocal tension membranes, the meninges that separate the anatomical areas of the central nervous structures; the tentorium separates the brain from the cerebellum (Figure 3) [22].

The tentorium cerebelli is located in the posterior cranial fossa with a semilunar shape; it is a transverse septum or tentorial diaphragm. It covers the cerebellum and acts as a support for the occipital lobes of the brain mass. Above the supratentorial portion and in the center, the falx of the brain merges, to continue below with the falx of the cerebellum [22]. The anterior portion is concave while its posterior area is convex; the tentorium splits and involves in its path the upper edge of the petrous portion of the temporal bone and posteriorly the squama of the occipital bone and part of the parietal bone, including the upper petrous sinus and the transverse sinus, respectively [22]. From the outside, we can delimit the tentorium cerebelli with our fingers, as it is possible to imagine a line (right and left) that starts from the external occipital protuberance up to asterion, a small depression that delimits the intersection of the temporal, parietal and occipital bones. The supratentorial innervation is provided by the nervus tentorii, an ophthalmic branch of the trigeminal nerve [22]. The subtentorial area is affected by the spinal nerves (C1-C3), which pass through the foramen magnum of the occipital bone and hypoglossal holes, and by the tenth cranial or vagus nerves and twelfth cranial or hypoglossal [22]. The sympathetic system innervates the above and subtentorial portion via the cervical perivascular plexuses; animal studies show a direct relationship with the stellate ganglion from the thoracic outlet [22].

Systemic myofascial relationships of the five diaphragms: posterolateral area

**Figure 4:**
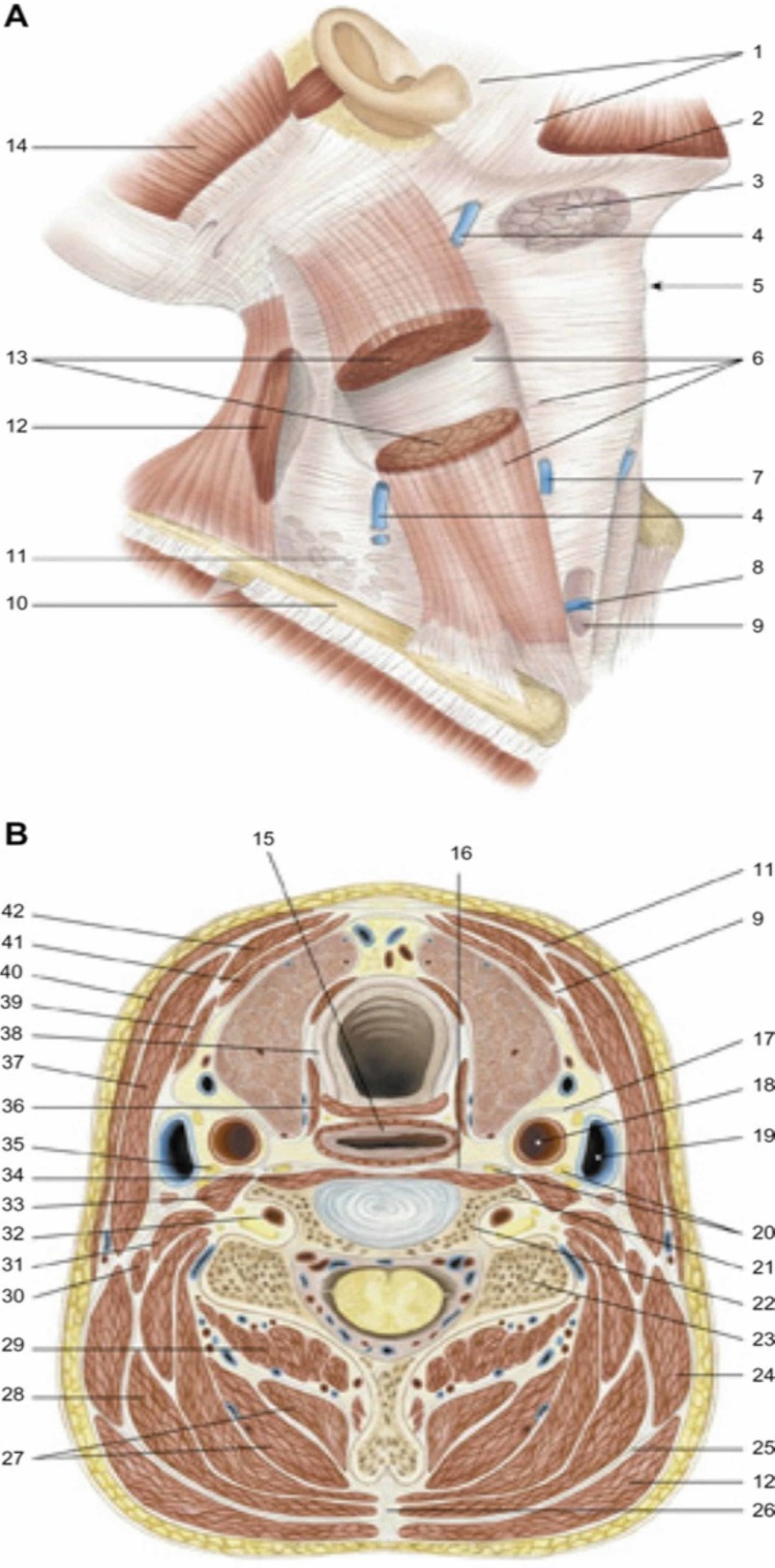
Fascias of the neck 1: parotid fascia, masseteric fascia; 2: platysma; 3: submandibular gland; 4: external jugular vein; 5: laryngeal prominence; 6: superficial cervical fascia; 7: anterior jugular vein; 8: the jugular venous arch; 9: middle cervical fascia; 10: clavicle; 11: superficial cervical fascia; 12: trapezius muscle; 13: sternocleidomastoid muscle; 14: occipital muscle (of the epicranic muscle); 15: esophagus; 16: deep cervical fascia; 17: carotid sheath; 18: common carotid arter; 19:  internal jugular vein; 20: chain of the sympathetic nervous system; 21: 6° cervical vertebrae, anterior tubercle; 22: 6° cervical vertebrae, transverse process; 23: 6° cervical vertebrae, posterior tubercle; 24: levator scapula; 25: nuchal’s fascia; 26: nuchal ligament; 27: semispinalis muscle of the head and neck; 28: splenius muscle of head and neck; 29: multifidus muscle of the neck; 30: posterior scalene muscle; 31: middle scalene muscle; 32: vertebral artery; 33: anterior scalene muscle; 34: long muscle of the neck; 35: phrenic nerve; 36: inferior constrictor muscle of the pharynx, cricopharyngeal portion; 37: sternocleidomastoid muscle; 38: trachea; 39: omohyoid muscle; 40: platysma; 41: muscle sternothyroidean; 42: muscle sternohyoidean. Reproduced with permission Anastasi et al. AA VV, Anatomia dell’uomo, fourth edition, 2010, pp 162. Editor: Edi-Ermes, Milano [Human Anatomy].

The whole human body is a network of connections, a functional continuum, a holobiont that interacts with the inside and the outside as a unit [23-24]. The posterolateral areas of the five diaphragms are connected by a myofascial system (connective and muscle tissue): the thoracolumbar fascia. The latter is a complex structure of muscles and ligaments comprising the nuchal area up to the lower limbs and from the surface up to the spine [25]. The sub-occipital muscles (rectus capitis posterior minor or RCPmi, rectus capitis posterior major or RCPma, oblique capitis inferior or OCI, oblique capitis superior or OCS) are linked to the dura mater. In particular, the first three of these muscles form a myodural bridge, which connects them to the subtentorial dura mater (cerebellar falx) [26]. The suboccipital muscles are part of the deep muscles of the thoracolumbar fascia [25]. The nuchal ligament (NL), part of the trapezius muscle and the superficial thoracolumbar fascia, has a relationship with the dura mater through a fascia (dense and fibrous structure) called To Be Named Ligament (TBNL); TBNL, consisting of arcuate or radiated fibers, originates from the posterior and inferior edge of the NL [26]. TBNL collaborates in the formation of the myodural bridge and the occipital muscles themselves; before the myodural bridge, they send connective tissue fibers to the TBNL [26]. Throughout the dural tract of the spine, including the cervical tract and in the suboccipital area, we can find the denticulate ligaments or Hoffman's ligaments. These ligaments at the cervical level have a caudocranial direction and have a close relationship with the posterior longitudinal ligament (PLL) and with the spinal roots, with which roots form a functional system (nerve roots and denticulate ligaments or NRDL) [27]. PLL is fused with the periosteum of the vertebrae (from the basioccipital area to the coccyx) and venous pathways pass through its fibers which will drain into the anterior internal vertebral plexuses [28]. PLL and NRDL constitute a system that can uniquely influence the dural and muscular system, as the movement of the vertebrae stimulates the change of myofascial and fascial tension (muscles and ligaments). The ligamenta flava has a close relationship with the dural tissue at the cervical level and throughout the vertebral tract, through the posterior epidural ligaments (PELs) [29]. The suboccipital muscles merge with the occipitofrontalis or epicranius muscle; this muscle covers the occipital-parietal-frontal area, through a muscular area (occipital and frontal) and an aponeurosis below the galea capitis or aponeurotic galea [7]. The occipitofrontalis is innervated by the extracranial portion of the facial nerve (auricular nerve); it merges with the temporoparietalis muscle, the levator muscle of the eyelid and the Müller's muscle [30-31]. The levator muscle and Müller's muscle merge with the extrinsic muscles of the eye via the fascia of Tenon [2]. From an embryological point of view, the musculature of the tongue derives from the occipital area and in adults we find these occipital-cervical relationships in the suprahyoid area, including the perivertebral spaces [19]. The interpterygoid fascia starts from the base of the skull with a medial vector, covering the oval foramen and the sphenoid spine, involving the tympanosquamous suture and the sphenopetrosal fissure [32]. The interpterygoid fascia covers the anterior surface of the styloid process, merging with the styloglossus muscle (part of the extrinsic musculature of the tongue) and with other muscles such as the styloid and stylopharyngeal muscle, the latter two fundamentals for the functioning of the tongue [32-33]. The tensor-vascular styloid fascia (from the lower limit of the tensor veli palatine muscle to the styloid process) laterally covers the styloid prominence and merges, finally, into the fascial network of the internal carotid artery [32]. The stylopharyngeal fascia merges into the fascia of the internal carotid artery along with the fascia of the capitis lateralis muscle and the fascia of the digastric muscle [32]. The interpterygoid fascia involves the fascial system of the internal carotid anterolaterally, where different fascial structures converge [32]. The palatoglossus muscle is in continuum with the fibers of the superior pharyngeal constrictor muscle and the pharingobasilar fascia; the latter starts from the pharyngeal tubercle of the occipital bone and merges with the buccopharyngeal or visceralis fascia [34-35]. The visceralis fascia covers the pharyngeal muscles and other visceral structures of the neck (pharynx, esophagus, larynx, thyroid) [35]. The intercarotic fascia or alar fascia involves the visceralis fascia in its path [35]. The retropharyngeal bands (visceral, alar and prevertebral fascia) are in communion with the posterolateral muscles of the neck (longus capitis and longus colli, scalene, levator scapulae) through the prevertebral fascia; the prevertebral fascia can merge with the anterior longitudinal ligament (ALL) [35]. The alaris fascia (not to be confused with the alar fascia) extends from the base of the skull to the last cervical vertebrae in a caudal direction and is found between the carotid fascia and the prevertebral fascia [35]. The connective tissue layer that covers the NL or superficial fascia of the neck envelops the neck and inserts on the hyoid bone, up to the lower surface of the mandibular bone; in its path, it wraps the stylohyoid and digastric muscles, the trapezius and sternocleidomastoid muscles (SCM), the mylohyoid muscle or buccal floor and the mastoid processes of the occipital bone [36]. The point of contact between the superficial and deep layer of the fascial continuum is referred to as the superficial muscular and aponeurotic system (SMAS), with dense and fibrous adhesions between the two layers at the level of the parotid and preauricular portion [37]. The deep layer involves the remaining deeper neck muscles (anterior and posterior), the NL, the PLL and the ALL, the anterior scalene, starting from the base of the skull (Figure 4) [37].

The thoracic outlet or upper thoracic diaphragm is in myofascial continuity with the tentorium and the lingual complex through some structures, such as the trapezius muscle and all the deep muscles of the cervical tract; the superficial and deep muscles of the posterior column fall within the system of the thoracolumbar fascia [25]. The cervical posterior superficial fascial layer continues with the trapezius muscle, overcoming the supraclavicular triangle, and involves the clavicle, acromion and the spine of the scapula [36]. The posterior cervical layer merges with the superficial layer at the level of the scapula, merging with the connective tissue of the subclavian artery [36]. The prevertebral fascia covers the deep fascia and divides at the level of the carotid tubercle or Chassaignac tubercle at the height of the sixth cervical vertebra; the fascia follows the deep muscles (medial to the longus colli and the lateral portion of the anterior scalene), crosses the lateral cervical triangle through the posterior interscalene space [35]. When the prevertebral fascia divides, it comes into contact with the epidural space (between the yellow ligament and the dura mater), creating another important dural contact site; the prevertebral fascia continues its work of connection between the tentorium cerebelli, the lingual complex, the thoracic outlet [35]. Through the ALL, the prevertebral fascia touches the suprapleural membrane or Sibson fascia, while laterally it merges with the fasciae of the axilla creating the axillary ligament or axillary arch or Langher arch [38]. The connective tissue that surrounds each structure not only brings together every anatomical aspect (solid and liquid fascia) but this fascial continuum allows the movement of the different structures and the transmission of innumerable biochemical and mechanometabolic messages [24]. The hyoid bone plays an important role in that it connects the base of the skull, the tongue and the buccal floor, and the shoulder girdle (thoracic outlet); the omohyoid muscle connects the myofascial infrahyoid portion, the scapula, and the posterior portion of the thoracolumbar fascia. The omohyoid muscle can also arise from the mastoid process of the temporal bone and merge with the SCM muscle in the clavicular portion, creating the sternocleid-omomastoid muscle or affect the hyoid bone and clavicle; it can arise from the transverse process of C6 or come into contact and then merge with the sternohyoid muscle or in rare cases, it may be absent [39]. Usually, the omohyoid muscle runs posterior to the SCM muscle and passes over the internal jugular vein. The infrahyoid muscles are surrounded by the deep fascial layer, which layer touches the SCM muscle laterally [40]. The fasciae of the cervical tract will form the various connective layers that relate the diaphragm muscle and the previous diaphragms [2, 7]. The deep fascia of the neck when it reaches the thoracic outlet divides, wrapping the intercostal muscles and the internal thoracic chest (endothoracic fascia); the latter is in contact with the parietal pleura [41]. The endothoracic fascia is in communication with all the viscera of the mediastinum through the visceral fascia. The viscera of the mediastinum are covered by a visceral fascia deriving from the deep fascia of the neck: the fascia covering the parietal pleura communicates with the parietal pericardium; the Morosow fascia or interpleural ligament connects the two lungs posteriorly; the esophagus and aorta communicate with the two lungs via fascial ramifications of the meso-esophagal fascia; the latter also connects the bronchi, the parietal pericardium and the trachea [41]. The broncho-pericardial or tracheobronchial-pericardial fascia connects the bronchi and the parietal pericardium in the area of the left atrium; the pretracheal anterior fascia (originates from the thyroid cartilage merges with the posterior portion of the pericardium and the endothoracic fascia that covers the diaphragm muscle [41]. The parietal pericardium touches the posterior endothoracic fascia of the sternal body and some ribs (fourth to the sixth in the left area), the endothoracic fascia that covers the diaphragm muscle or the phrenopericardial ligament; it continues posteriorly to merge with the endothoracic fascia at the level of D10-D11, enveloping the aorta and esophagus [41]. The visceral fascia that covers the bases of the lungs merges with the endothoracic fascia that lines the diaphragm; the triangular or inferior ligaments of the lung (created by the visceral and parietal fascia) merge with the endothoracic fascia [41]. The membrane of Laimer or phrenoesophageal membrane (above and below the respiratory diaphragm), involves the passage of the esophagus at the level of the esophageal hiatus; this membrane merges with the endothoracic fascia [10]. Below the diaphragm and in communication with the esophagus we find the muscle of Low and the transverse intertendinous muscle, in conjunction with the fascia transversalis which covers the lower portion of the diaphragm and which fascia derives from the endothoracic fascia [10]. The Hilfsmuskel muscle derives from the area of the esophageal hiatus below the diaphragm muscle, connecting to the celiac trunk or to another vascular structure such as the superior mesenteric artery; the Hilfsmuskel muscle continues with a connective bridge to connect and merge with the retro-pancreatic fascia or Treitz's fascia or suspensory muscle of duodenum, which last connects the upper area of the duodenum [10]. The fascia covering the lower diaphragm (fascia transversalis) merges with some viscera via connective tissue connections or fascial ligaments. Glisson's capsule involves a large part of the diaphragm muscle, the phrenic-gastric ligament (connects the fundus of the stomach), the phrenic-colic ligaments (connects the ascending colon to the right and descending to the left), hepatic ligaments (coronary ligament, falciform ligament, triangular ligaments) [7]. Anteriorly, the lateral pillars merge with the epimysium of the psoas and quadratus lumborum muscles while, posteriorly, they merge with the thoracolumbar myofascial complex [7]. From the lateral pillars and precisely from the twelfth rib, the lateral raphe arises, a connective portion that is part of the thoracolumbar continuum, which raphe inserts above the iliac crest [7]. The transversalis fascia covers the viscera of the pelvic space transforming into an endopelvic fascia; the latter is divided into parietal and visceral fascia [7]. The endopelvic fascia covers the muscles that form the pelvic floor (levator ani and the ischiococcygeus muscle), the internal obturator and the piriformis muscle; finally, it merges with the presacral and periosteal fascia of the pubic area [42]. In the path of the endopelvic fascia, we find other small portions of connective tissue such as the Denonvilliers fascia (between the rectum and the seminal vesicles in men or between the rectum and vagina in women or the rectogenital fascia), the Walderyer fascia (between the posterior portion of the rectum at the caudal level of the sacrum and the presacral fascia or rectosacral fascia) [43]. The transversalis fascia comes into contact with the Gerota fascia or renal fascia, which covers the kidneys and adrenal glands; the transversalis fascia comes into contact with the Toldt fascia (fascia that covers the Gerota fascia anteriorly), which expands to involve many abdominal and pelvic viscera, to merge with the endopelvic fascia and the Fredet fascia (between the pancreatic-duodenal visceral peritoneum and the ascending mesocolon) [44]. The fascial system also involves all visceral ligaments (periurethral, paraurethral and pubourethral and genital ligaments) and all somatic ligaments such as pubic ligaments (arcuate pubic, superior pubic), sacral ligaments (sacrotuberous, sacrospuberous, sacrospinous, long posterior sacroiliac and short posterior sacroiliac, anterior sacroiliac and sacroiliac interosseous) [45]. The thoracolumbar fascia that communicates with the muscular portion of the respiratory diaphragm posteriorly, continues, involving the lumbosacral and posterior pelvic muscle area (biceps femoris, piriformis, gluteus maximus, multifidus, longissimus thoracis, iliocostalis lumborum, erector spinae) [45-48]. The posterior-lateral myofascial continuum connects all the mentioned diaphragms, constituting an important tool for osteopathic medicine for the resolution of dysfunction or the maximum clinical help that a patient can obtain [2, 49-50].

## Conclusions

The article reviewed the myofascial relationships of the diaphragms considered by osteopathic medicine, such as the respiratory diaphragm muscle, the tentorium cerebelli, the lingual complex, the thoracic outlet and the pelvic floor. The text briefly reviewed the anatomy and innervation of the individual diaphragms. The text is updated and with anatomical information not included in the previous single article.
